# Serum Lipid Profile in Subjects with Traumatic Spinal Cord Injury

**DOI:** 10.1371/journal.pone.0115522

**Published:** 2015-02-23

**Authors:** Martin Laclaustra, Elizabeth Louise Maayken Van Den Berg, Yamilée Hurtado-Roca, Juan Manuel Castellote

**Affiliations:** 1 Department of Epidemiology, Atherothrombosis and Imaging, Centro Nacional de Investigaciones Cardiovasculares (CNIC), Madrid, Spain; 2 Department of Preventive Medicine and Public Health, School of Medicine, Universidad Autonoma de Madrid, Madrid, Spain; 3 Department of Epidemiology, St. Louis University, St. Louis, Missouri, United States of America; 4 Department of Rehabilitation, Aged and Extended Care, School of Health Sciences, Flinders University, Adelaide, Australia; 5 National School of Occupational Medicine, Carlos III Institute of Health, Madrid, Spain; 6 Department of Physical Medicine and Rehabilitation, School of Medicine, Complutense University of Madrid, Madrid, Spain; Heidelberg University Hospital, GERMANY

## Abstract

**Background and Aims:**

Few large studies have examined the relationship between spinal cord injury (SCI) and lipid profile. We studied serum lipid concentrations in subjects with traumatic SCI in relation to the degree of neurological involvement and time since injury, and compared them with values from a reference sample for the Spanish population (DRECE study).

**Materials and Methods:**

A retrospective cohort was built from 177 consecutive cases with traumatic SCI admitted to the SCI unit of the Miguel Servet Hospital in Aragon (Spain). Outcome measures (cholesterol, triglycerides, HDL-c and LDL-c levels) were analyzed according to the ASIA Impairment Scale (AIS), neurological level of injury (involvement of all limbs vs. only lower limbs), and time since injury. All analyses were adjusted for age and sex.

**Results:**

Cases without preserved motor function (AIS A or B) had lower total and HDL cholesterol than the others (-11.4 [-21.5, -1.4] mg/dL total cholesterol and -5.1 [-8.8, -1.4] mg/dL HDL-c), and cases with all-limb involvement had lower total, HDL, and LDL cholesterol than those with only lower-limb involvement (-14.0 [-24.6, -3.4] mg/dL total cholesterol, -4.1 [-8.0, -0.2] mg/dL HDL-c, and -10.0 [-19.7, -0.3] mg/dL LDL-c) (all p<0.05). No association was found between lipid concentrations and time since injury. Concentrations of lipid subfractions and triglycerides in SCI subjects were lower than in sex- and age-stratified values from the reference sample.

**Conclusion:**

A high degree of neurological involvement in SCI (anatomically higher lesions and AIS A or B) is associated with lower total cholesterol and HDL-c.

## Introduction

Most people with spinal cord injury (SCI) perform less physical activity[1], have less social interaction[2,3], and maintain fewer active muscle groups than the general population. SCI affects well-being, morbidity, and life expectancy. Associated secondary diseases derive not only from the primary damage but also from the reduced mobility. SCI patients die prematurely, underlining the need to study the factors affecting their survival. They have higher cardiovascular disease incidence[4], and decreased levels of high density lipoprotein cholesterol (HDL-c) have been described. Only a few studies have examined the lipid profile in SCI using a design that includes controls and a moderately sized sample[5–9]. Moreover, only large reference clinical centers can include enough cases to cover different motor function grades, neurological levels of injury, and elapsed times since injury. The lipid profile of SCI cases in a large sample was explored by Bauman et al.[10], but data were not compared with values from a reference group.

We measured serum lipids in a sample of 177 consecutive cases of SCI treated at a reference center. The sample size allowed study of the degree of neurological involvement, adjusting for age and sex. We also compared serum lipids from SCI cases with established normal values for the Spanish population.

## Materials and Methods

### Study Design and Subjects

A retrospective cohort was built from the records of 177 consecutive traumatic SCI cases who received routine annual follow-up care at Miguel Servet Hospital, site of the only specialized SCI unit in Aragon, Spain (population 1,277,471 as of 1/1/2006). SCI subjects whose SCI injury is stable are treated and followed-up primarily in the region where they live. This study focused on cases of traumatic SCI, where the neurological condition remains fairly stable after the acute phase and the time elapsed since injury is known. Data were included from subjects aged between 15 and 60 years of age at least one year after injury. The main causes of traumatic SCI in these patients were motor vehicle accidents and accidental falls. Patients with malignant or concurrent diseases affecting nutrition or lipid profile were excluded.

This study was approved by the Health Research Ethics Board (Approval number (2008/01). Since all identifying personal information was removed from the secondary files prior to analysis, the review board waived the requirement for written informed consent from the patients involved.

### Clinical and laboratory data

Data related to age, sex, date of injury and neurological characteristics were obtained from medical records at the hospital’s SCI unit. SCI diagnosis was established according to the diagnostic codes of the International Classification of Diseases, Ninth Revision, Clinical Modification (ICD-9-CM). Laboratory fasting blood sample data were obtained during annual visits in 2005 and 2006. Cholesterol, triglycerides, and HDL-c were measured by standard enzymatic methods. Low density lipoprotein cholesterol (LDL-c) was calculated with the Friedewald formula.

The following neurologic characteristics were assessed from the patients: motor function grade using the American Spinal Injury Association (ASIA) impairment scale (AIS), neurological level of injury, and limbs involvement (upper and lower limbs or lower limbs only).

### Statistical analysis

Results are presented as means and standard deviations (SD) for quantitative data or as percentages for qualitative data.

Motor function grades (AIS) divided the sample unevenly, and cases were grouped into motor-complete lesions (no preservation of motor function; AIS A or B) versus the rest. Neurological level of injury (classified as cervical, thoracic, or lumbosacral) was further regrouped according to whether damage was proximal (upper limbs affected in addition to lower limbs and trunk) versus distal (only lower limbs affected).

Linear regression models were used to estimate the differences in each lipid parameter among the classifying groups (motor grade, neurological level, and time since injury), after adjustment for age (10-year categories) and sex. The models built to assess the effect of the degree of neurological involvement on lipids included motor grade, neurological level, age, and sex, so that estimates for motor grade and neurological level are mutually adjusted. An initial model included motor grade and neurological level as dichotomous variables (motor grade: AIS A-B vs. AIS C-E; neurological level: proximal damage vs. distal damage); a second model detailed neurological level as cervical, thoracic, or lumbosacral, and a third considered four groups according to the combination of motor grade and dichotomous classification of neurological level. To assess the effect of time since injury the models included time, age, and sex.

To assign an age-and-sex-adjusted Z-score to each case, we compared outcomes with serum-lipid age-and-sex-stratified reference percentile tables of the Spanish population from the DRECE study[11]. Mean score values were calculated to reflect the differences between the SCI sample and the Spanish population. To aid interpretation, we converted scores into estimated differences in mg/dL by multiplying each score by the corresponding standard deviation in the Spanish population[11].

Differences were considered significant when p≤0.05 and confidence intervals (CI) did not include zero.

## Results

The mean age of the 177 traumatic SCI cases was 40.6 (9.9) years and most cases were men (133, 75.1%). Within the cohort, 113 (63.9%) had an AIS A or B score and the average time since injury was 13.6 (8.1) years (Table 1).

**Table 1:** Sample characteristics.

		**Neurological level of injury**		
	**Overall**	**Cervical**	**Thoracic**	**Lumbosacral**
**N**	177	52	91	34
**Age** *	40.6(9.9)	40.9(10.1)	40.7(9.4)	40.1(11.0)
**Male** †	75.1	78.9	76.9	64.7
**Time since injury** *	13.6(8.1)	13.0(8.5)	14.7(8.1)	11.6(7.1)
**AIS A-B** †	63.9	42.3	85.7	38.2
**Cholesterol** ‡	183.1(35.1)	175.5(36.8)	185.6(32.6)	187.7(38.3)
**Triglycerides** ‡	94.9(58.9)	92.8(60.6)	101.8(65.3)	79.7(28.7)
**HDL-c** ‡	42.6(12.7)	40.5(14.3)	41.7(11.2)	48.3(12.4)
**LDL-c** ‡	121.5(30.8)	116.5(32.7)	123.6(29.3)	123.5(31.7)

Notes: AIS, ASIA Impairment Scale.

The data are means (SD) for quantitative variables and percentages for qualitative variables.

*, years

†, %

‡, mg/dL

### Serum lipid differences according to neurological involvement and time since injury

We compared cases with no motor preservation (AIS A-B) (n = 113) versus the rest (n = 64), adjusting for age, sex, and level of injury. AIS A-B cases showed statistically significant lower levels of total cholesterol [-11.4 (-21.5, -1.4) mg/dL] and HDL-c [-5.1 (-8.8, -1.4) mg/dL] than those with motor preservation (all p<0.05, Table 2). AIS A-B patients also showed a tendency to have lower LDL-c and higher triglyceride levels.

**Table 2:** Differences in serum lipids classified according to motor grade and neurological level of injury.

	**Motor grade**			**Neurological level of injury**		
	AIS A-B minus AIS C-E		p	Proximal minus distal damage		p
	(n = 113)	(n = 64)		(n = 52)	(n = 125)	
Cholesterol *	-11.4	(-21.5, -1.4)	0.026	-14.0	(-24.6, -3.4)	0.010
Triglycerides *	11.9	(-6.1, 29.9)	0.194	0.3	(-18.6, 19.2)	0.974
HDL-c *	-5.1	(-8.8, -1.4)	0.007	-4.1	(-8.0, -0.2)	0.037
LDL-c *	-8.7	(-17.9, 0.5)	0.064	-10.0	(-19.7, -0.3)	0.044

Notes: AIS, ASIA Impairment Scale.

All data shown are adjusted differences (95%CI) in a regression model including motor grade (AIS A-B vs. C-E), neurological level of injury (proximal [upper limb involvement] vs. distal [only lower limb involvement]), age, and sex.

*, mg/dL

Neurological level of injury was considered after adjustment for motor grade, age, and sex. Individuals with proximal damage (with upper limb involvement) had lower total cholesterol [-14.0 (-24.6, -3.4) mg/dL], lower HDL-c [-4.1 (-8.0, -0.2) mg/dL], and lower LDL-c [-10.0 (-19.7, -0.3) mg/dL] compared with those with distal damage (only lower limb involvement) (all p<0.05, Table 2). A more detailed analysis considering cervical, thoracic, and lumbo-sacral cases separately revealed that the main differences were present at the cervico-thoracic transition (Table 3). Statistically significant differences in total cholesterol, HDL-c, and LDL-c were also found when comparing patients with the most severe condition (AIS A-B individuals with proximal damage) with those with the closest-to-normal condition (individuals with an AIS C-E score and only distal damage) (Table 4).

**Table 3:** Differences in serum lipids classified according to detailed neurological level of injury.

	**Cervical**	**Thoracic**	**Lumbo-sacral**	**p trend**
**N**	52	91	34	
Cholesterol *	-11.1 (-24.9, 2.6)	4.5 (-9.2, 18.2)	0 (Reference)	0.055
Triglycerides *	10.3 (-14.1, 34.8)	15.7 (-8.7, 40.0)	0 (Reference)	0.513
HDL-c *	-5.6 (-10.6, -0.6)	-2.4 (-7.4, 2.6)	0 (Reference)	0.023
LDL-c *	-7.6 (-20.2, 5.0)	3.7 (-8.8, 16.3)	0 (Reference)	0.145

Notes: All data shown are adjusted differences in a regression model including motor grade (AIS A-B vs. C-E), neurological lesion level (cervical vs. thoracic vs. lumbo-sacral), age, and sex.

*, mg/dL.

**Table 4:** Differences in serum lipids classified according to a combined classification of motor grade and neurological level of injury.

	**AIS A-B & proximal damage**	**AIS C-E & proximal damage**	**AIS A-B & distal damage**	**AIS C-E & distal damage**
**N**	22	30	91	34
Cholesterol *	-27.3 (-44.1, -10.6)	-6.7 (-22.0, 8.6)	-6.7 (-19.1, 5.6)	0 (Reference)
Triglycerides *	11.8 (-18.2, 41.9)	1.7 (-25.8, 29.2)	12.8 (-9.4, 34.9)	0 (Reference)
HDL-c *	-9.3 (-15.4, -3.1)	-3.9 (-9.5, 1.7)	-5.0 (-9.5, -0.4)	0 (Reference)
LDL-c *	-20.4 (-35.8, -5.1)	-3.1 (-17.2, 10.9)	-4.3 (-15.6, 7.1)	0 (Reference)

Notes: AIS, ASIA Impairment Scale.

All data shown are adjusted differences in a regression model including the combined motor grade and neurological lesion level, age, and sex.

*, mg/dL.

After adjusting for age, there was no significant relationship between serum lipid values and time since injury, whether stratified into 5-year groups or collapsed into a dichotomous variable of below or above than 10 years (S1 Table).

### Serum lipid differences between SCI cases and the reference population

Age- and sex-adjusted Z-scores for lipid values in SCI cases with respect to the reference Spanish population revealed below-normal lipid concentrations for all fractions (Table 5). Conversion to the equivalent values in mg/dL confirmed that SCI cases had lower serum concentrations of all lipids than the general population: -29.3 mg/dL cholesterol, -58.2 mg/dL triglycerides, -10.4 mg/dL HDL-c, and -4.6 LDL-c. These differences were more pronounced for the most severe condition (AIS A-B individuals with proximal damage) (Table 5 and S2 Table).

**Table 5:** Serum lipid values comparing SCI subjects with age- and sex-stratified reference values for the general population.

	**General Population**	**Overall SCI sample vs. general population (n = 177)**		**AIS A-B & proximal damage vs. general population (n = 22)**	
	mean (SD) mg/dL	z-score	mg/dL	z-score	mg/dL
**Cholesterol**	191 (43.6)	-0.67 (-0.80, -0.54)	-29.3 (-34.9, -23.7)	-1.22 (-1.58, -0.85)	-53.2 (-69.1, -37.2)
**Triglycerides**	104.6 (86.1)	-0.68 (-0.83, -0.52)	-58.2 (-71.4, -45.1)	-0.76 (-1.37, -0.16)	-65.6 (-117.7, -13.5)
**HDL-c**	55.1 (13.7)	-0.76 (-0.90, -0.61)	-10.4 (-12.4, -8.4)	-1.25 (-1.68, -0.82)	-17.2 (-23.1, -11.3)
**LDL-c**	115.6 (37.9)	-0.12 (-0.25, 0.01)	-4.6 (-9.6, 0.4)	-0.52 (-0.99, -0.05)	-19.9 (-37.7, -2.1)

Notes: AIS, ASIA Impairment Scale.

Mean Z-scores were multiplied by the general population SD to obtain estimates in mg/dL for easier interpretation of the data.

## Discussion

In this study of consecutive SCI cases admitted to a rehabilitation facility, we show that serum total cholesterol and HDL-c are lower when the subject has a lower motor grade and a higher neurological level of injury. These measures of neurological involvement affect lipid levels independently. Time since injury did not influence the lipid profile at the medium or long term periods studied, which suggests that lipid levels change early, within the first 5 years after injury. The observed differences were consistent for comparison of different grades within our sample and for comparison of the whole sample with the general population.

The high prevalence of cardiovascular disease in paraplegic SCI subjects[12] has prompted calls for the implementation of preventive measures[13,14]. Cardiovascular disease is responsible for many deaths in SCI subjects, and cardiovascular deaths occur earlier in life than in the general population[4]. Survival is also lower than in the general population, with tetraplegic SCI individuals more affected than paraplegic ones[15]. The evidence does not identify SCI as an independent cardiovascular risk factor[5]; rather, SCI is likely to aggravate conventional risk factors. Our study demonstrates that serum lipid values are altered after SCI in relation to the degree of neurological involvement. Given that the pro-atherosclerotic lipid profiles of SCI patients may place them at elevated risk of cardiovascular disease, this finding warrants longitudinal studies to determine the relative importance of individual cardiovascular risk factors in the survival of SCI populations. Bauman et al.[10] obtained similar findings in a larger sample; however, differences in the statistical adjustments performed make it difficult to compare those findings with the present study, and the Bauman study moreover did not compare serum lipids from SCI cases to a reference group.

High serum cholesterol, in particular LDL-c, increases cardiovascular risk[16]. Interestingly, the SCI subjects in this study have lower total cholesterol than the general population. However, the difference was moderate, and might signify only a modest reduction in risk. Indeed, serum HDL-c, which associates inversely with risk[16], also decreased with increasing neurologic involvement among SCI patients, and was significantly lower compared with the general population. The National Cholesterol Education Program Adult Treatment Panel III (NCEP ATP III) criteria[16] define three bands of risk for HDL-c, with the middle band having a width of 20 mg/dL. The lipid differences found for HDL-c might therefore signify a substantial increase in cardiovascular risk. Overall, this mixed modification pattern might explain why the cardiovascular risk associated with SCI has remained unresolved. Variations in the metabolic changes for a particular SCI patient or for certain SCI subgroups could result in a normal or elevated cardiovascular risk, thus producing mixed research results[6,8,17].

The International Standards for Neurological Classification of Spinal Cord Injury (ISNCSCI) include the ASIA Impairment Scale (AIS), which, like the previous ASIA scale, differentiates several grades of motor function preservation. In the present study, lower motor grades (AIS A and B) were associated with lower total cholesterol and HDL-c, independently of the neurological level of injury. Motor grade association with serum lipid values is consistent with the changes in lipid levels in relation to the neurological level of injury. Indeed, the more detailed analysis according to neurological level confirmed marked differences associated with the cervico-thoracic transition: individuals with SCI at the thoracic or lumbo-sacral level, who are able to exercise with full use of upper limbs and some use of the trunk, maintain higher HDL-c values than patients with cervical injury. These findings are consistent with reports by Krum et al.[6], who related decreased HDL-c to low levels of physical exercise, and Bauman et al.[7,8], who found lower HDL-c concentrations in two series of SCI patients compared with able-bodied controls. To explore the underlying causes of the lipid profile of SCI patients, further studies are warranted into lifestyle and diet in SCI populations, including detailed surveys of nutritional laboratory markers and their relation to lipids. A higher neurological level of injury is clearly associated with an increase in subclinical atherosclerosis[18], but some studies[6,8] found no association with the serum levels of HDL-c and cholesterol, possibly reflecting differences in methodology and source populations.

All lipid fractions except for LDL-c were significantly lower in the SCI sample than in the general Spanish population[11]. These results are in agreement with Bauman et al.[7], who found lower levels of total cholesterol, HDL-c and LDL-c in a series of 320 SCI cases compared with a matched population, and Bauman et al.[8], where they reported lower levels of all serum lipids including triglycerides.

After adjusting for age, we found no association between serum lipids and time since injury grouped into five-year strata, which was the maximum reasonable disaggregation that our data allowed. Reductions of serum lipids occur acutely soon after injury and partially recover during the first year[19]; however, there is little information available on long term changes in serum lipids post injury. In the Bauman series[7,8] the average time since injury was close to 16 years, but the authors did not analyze subgroups according to time since injury. Krum et al.[6] also found low HDL-c values at 10 years after injury in a series of 327 cases. However, they did not report whether values in the group with more than 10 years follow-up differed from those in the group with more recent injury. Sabour et al.[20] reported that waist circumference, a parameter inversely related to HDL-c, increases with time since injury. Our results show little influence of time since injury over periods of more than 5 years, and suggest that lipids change within the first years after the injury and thereafter remain stable.

Three mechanisms, two physiological and one nutritional, might explain the association between the degree of neurological involvement and lipid changes in SCI patients. First, physical inactivity, as a consequence of limited mobility, is among the leading causes of low HDL-c[16] and has demonstrated negative health consequences in SCI subjects[21,22]. In addition to sedentary lifestyle, SCI subjects have lower metabolic rates, explained by a reduction in the muscle mass able to exercise and a reduced fat-free body mass due to muscular atrophy[23,24]. The second possible mechanism is related to dysfunction of the autonomic nervous system[25–27]. After injury, sympathetic tone diminishes, reducing the resting metabolic rate, which together with increased leptin levels could increase the risk of obesity[28]. Although no studies have specifically related autonomic disorders in SCI to serum lipid changes, the close interaction between adipose tissue and the nervous system is a plausible cause of the observed association[19,29,30]. The potential nutritional mechanism is related to patients’ possible dependency on others for nourishment. Diet plays an important role in both the modification of serum lipids and the prevention of cardiovascular disease[16,31]. Achieving an appropriate diet is a challenge for SCI patients and often requires specialized assessment; risk of either undernutrition or overnutrition affects up to 90% of SCI patients[32]. The decreases in total cholesterol and LDL-c may indicate two very different situations: the consequence of a more carefully chosen diet by a nutritionist[16] or an inadequate coverage of nutritional needs. Similarly, HDL-c can also decrease due to malnutrition[33]. In contrast, overnutrition occurs when the adjusted diet does not include the sufficient caloric reduction required by these patients[34]. This energetic imbalance can lead to the atherogenic dyslipidemia of metabolic syndrome, which includes low HDL-c[30,31].

This study provides evidence that serum lipid concentrations are modified after SCI. Given the potential of serum lipid changes to increase cardiovascular risk in SCI patients, control of lipids should be included within preventive strategies in SCI healthcare programs. Possible approaches to addressing the detrimental effects on lipid concentrations described here include promoting adequate physical activity and exploring the potential benefits of neuromuscular stimulation[35]. Additionally, close monitoring of dietary needs and specialist-driven corrections in response to lipid profile changes could prevent the malnutrition that is another possible cause of altered lipids profiles in SCI patients[32].

### Study strengths and limitations

Important strengths of our study are the reasonably large series size, the homogeneous data measurement, and the consistent results across the parameters studied. Nonetheless, there are some limitations that should be considered. Owing to the observational nature of the study, the cases were not evenly distributed across the neurological levels of injury and AIS, precluding a detailed *per level* or *per grade* comparison. Subjects’ weight was not collected in all cases due to difficulties inherent to this population[36], and thus we were not able to discriminate between overnutrition and undernutrition, hampering clarification of the origin of the observed lipid differences. The main confounders, sex and age, were taken into account in the analysis, and SCI causes that can produce metabolic changes were excluded by design, as we only selected cases of SCI from traumatic causes. It is not possible to fully rule out contributions from unmeasured confounders that could influence the initial level of injury and motor grade and the observed lipid profile; however, these are unlikely to be present. Observation of long times since injury is necessarily more likely in older patients, and adjusting for age requires larger cohorts since there are very few young subjects with a comparably long elapsed time since injury. Moreover, our analysis could have been affected by survival bias. We have assumed that the reference values for the Spanish population from the DRECE study are representative of the population source for our patients (they were Spanish cases). If this is not the case, estimates from the comparison with the general population may be biased. We believe that our results are generalizable to the Spanish traumatic SCI patient collective. We collected all traumatic SCI cases that were followed up in routine annual check-ups at a specialized SCI Unit. This unit, one of 13 in Spain, covers a delimited health area, but we consider the results to be representative of the whole country, as shown in previous epidemiological studies by our research group[37,38]. It is clear that the detected differences between our cohort and the general population depend on the particular aggregate of grades and levels in the sample. Therefore, to extend generalizability, we performed additional per subgroup comparisons.

In conclusion, we have shown that the degree of neurological involvement in SCI subjects, whether considered as motor grade or neurological level, is associated with decreased levels of total cholesterol and HDL-c. In addition, SCI patients have lower serum levels of total cholesterol, triglycerides, HDL-c, and LDL-c than the general population. Taking into account the potential mechanisms underlying these lipid changes, SCI patients may need special preventive measures for cardiovascular disease. These would necessarily include regular screening and correction of lipid and non-lipid risk factors that might arise from the impaired motor function consequent to the injury, the hormonal changes related to the degree of neurological involvement, and the nutritional imbalance that is common among these patients.

## Supporting Information

S1 Table Differences in serum lipids classified according to the time since injury. All data shown are adjusted differences in a regression model including the time since injury (two groups), age, and sex.
(EPS)

S2 Table Serum lipid values comparing SCI subject groups classified according to motor grade and neurological level of injury with age- and sex-stratified reference values for the general population. Mean Z-scores were multiplied by the general population SD to obtain estimates in mg/dL for easier interpretation of the data.
(EPS)
